# Mediating impact of sleep quality and duration on the relationship between socio-economic conditions and pain

**DOI:** 10.1093/ije/dyaf176

**Published:** 2025-10-30

**Authors:** Saman Khalatbari-Soltani, Dusan Petrovic, Carlos de Mestral, Pedro Marques-Vidal, Fiona M Blyth

**Affiliations:** School of Public Health, Faculty of Medicine and Health, University of Sydney, New South Wales, Australia; ARC Centre of Excellence in Population Ageing Research (CEPAR), University of Sydney, New South Wales, Australia; Center for Primary Care and Public Health (Unisanté), Department of Epidemiology and Health Systems, University of Lausanne, Lausanne, Switzerland; Unit of Population Epidemiology, Division of Primary Care Medicine, Geneva University Hospitals, Geneva, Switzerland; Department of Medicine, Internal Medicine, Lausanne University Hospital (CHUV), and University of Lausanne, Lausanne, Switzerland; School of Public Health, Faculty of Medicine and Health, University of Sydney, New South Wales, Australia; ARC Centre of Excellence in Population Ageing Research (CEPAR), University of Sydney, New South Wales, Australia

**Keywords:** socio-economic determinants, pain, sleep, inequity, life course

## Abstract

**Background:**

Sleep-related problems exhibit strong social patterning and are associated with pain; however, their mediating role in socio-economic inequities in pain remains largely unknown. We aim to assess the extent to which life course socio-economic inequities in pain experience and severity are mediated through sleep quality and duration.

**Methods:**

We used data from 1719 individuals [52.0% women; mean age 60.1 years (SD 9.1)] from the Swiss CoLaus|PsyCoLaus study, with both sleep measures at baseline (2009–2012) and pain measures at follow-up (2014–2017). Life course socio-economic conditions were assessed by using the father’s and individual’s occupational position, educational level, and income. Potential mediators included self-reported sleep quality (measured by using the Pittsburgh Sleep Quality Index, ranging from 0 to 21, with high scores indicating poorer sleep quality) and duration (short <6 h/night; normal 6–8.5 h/night; long >8.5 h/night). Presence of pain, chronic pain, and pain severity were assessed by using a self-administered questionnaire. We estimated age, sex, and country of birth with adjusted ***β*** coefficient, odds ratio, and proportion mediated by using counterfactual mediation analyses.

**Results:**

Pain and chronic pain were reported by 47% and 40.8% of the participants, respectively. Among 699 participants with reported pain severity data, the mean pain severity score was 3.6 (SD 1.7) (scale ranging from 0 = no to 10 = worst imaginable pain). Sleep quality explained 30% of the educational, 21% of the occupational, and 45% of the income inequities in pain; 20% of the occupation and 51% of the income inequities in chronic pain; and 15%, 9%, and 16% of the father’s occupation, educational, and income inequities in pain severity, respectively. Short sleep duration explained 31% of the education inequities in pain and 37% of the education and income inequities in chronic pain.

**Conclusion:**

This study suggests poor sleep quality and short sleep duration partially mediate socio-economic inequities in pain. The role of other potential mediators at individual and societal levels should be further investigated.

Key Messages

- The extent to which sleep quality and duration mediate the association between life course socio-economic inequities and pain experience and severity was assessed.
- Sleep quality mediated ≤51% of the socio-economic inequities in pain, with the strongest mediation observed for income inequities in pain and chronic pain, and short sleep duration mediated 31% of the education inequities in pain and 36%–37% of the education and income inequities in chronic pain.
- Among socio-economically disadvantaged individuals, early intervention targeting sleep quality and duration may help reduce socio-economic inequities in pain, alongside tackling adverse socio-economic conditions as root causes.

## Background

Pain and pain-related diseases are among the leading cause of disability and disease burden worldwide [1–3]. With global population ageing, this burden continues to escalate, as pain prevalence peaks in late middle and older age [4, 5]. Disadvantaged socio-economic conditions have been associated with increased pain and worse pain-related outcomes [5–8]. Part of this social gradient can be explained through behavioural [9–11] or psychosocial mechanisms [11–17]. Unhealthy behaviours—more common among those with lower socio-economic conditions—can both contribute to and result from pain. Studies, mostly from Europe, show that behaviours such as smoking and physical inactivity partly explain these associations [9–11]. Psychosocial mechanisms have also been highlighted, including the roles of depression, anxiety, and stress [11–17].

Among the proposed underlying mechanisms, the effect of sleep-related disorders on pain has been a focus of growing interest [18, 19]. It has been previously shown that, compared with individuals in more socio-economically advantageous conditions, those with a lower educational level, occupational position, and economic difficulties are more likely to experience sleep problems [18, 20–24]. The relationship between sleep and pain has been described as bidirectional, though there is greater support for sleep impacting pain than vice versa [18, 25, 26]. Given the socio-economic inequities in sleep and sleep being a risk factor for pain, this highlights the importance of better understanding the extent to which the socio-economic inequities in pain are explained by sleep quality and duration. However, to date, only two studies—one from the UK and one from the USA—have evaluated the contribution of sleep to socio-economic inequities in pain as assessed by neighbourhood socio-economic conditions, reporting mixed results [12, 27]. Both studies are cross-sectional and use traditional mediation methods (e.g. difference methods) [12, 27], which limits understanding of the direction of associations and direct and indirect effect estimates.

No study to date has evaluated the mediating role of both sleep quality and duration—key risk factors for pain [18]—on socio-economic inequities in pain by using individual-level socio-economic indicators from both childhood and adulthood. This study aims to address this knowledge gap by using a counterfactual mediation model in a prospective cohort study from Switzerland.

## Methods

### Study population

We used data from the CoLaus|PsyCoLaus study—a population-based cohort of adults of European origin living in the city of Lausanne, Switzerland [28]. Briefly, a representative sample was collected through a simple, non-stratified random sampling of 19 830 individuals (35% of the source population) aged between 35 and 75 years. The study was initiated in 2003 (*n* = 6733), with the first follow-up conducted in 2009–2012 (*n* = 5064) and the second follow-up in 2014–2017 (*n* = 4881) [28]. For the present analysis, we used data from the first (when pain assessment was initiated) and second follow-up phases; the first follow-up is therefore considered the baseline for this study.

The inclusion criteria required participants to have both sleep and pain data. A total of 2586 participants completed measures of sleep at baseline, of whom 1719 also completed pain assessments in the follow-up (Supplementary Fig. 1). The analysis of chronic pain included 1687 participants, excluding 32 participants with missing chronic pain data. The analysis of pain severity included 699 participants who reported experiencing pain and had pain severity data at follow-up. For analyses related the participant’s own occupational position, analysis was restricted to 1066 participants who reported working at baseline. Supplementary Table 1 details the sample size and exclusion/inclusion criteria for each analysis.

### Exposures: socio-economic indicators

We used four indicators of socio-economic conditions assessed at baseline as the main exposure variables. Childhood socio-economic conditions, commonly indicated by the father’s occupational position, was classified into three main categories by using the European SocioEconomic Classification framework [29]: ‘high’ (higher professionals/managers, lower professionals/managers, higher clerical), ‘intermediate’ (small employers and self-employed, farmers, lower supervisors, and technicians), and ‘low’ (lower clerical, sales workers, skilled/unskilled workers). Adulthood socio-economic conditions included self-reported education, occupational position, and income. The highest level of attained education was classified as ‘high’ (university education), ‘intermediate’ (higher secondary education), and ‘low’ (lower secondary education or lower). Participants’ occupation was categorized as for the father’s occupation. Monthly household income before social charges had six response options used as income categories: >13 000, 9500–13 000, 7000–9500, 5000–7000, 3000–5000, and <3000 CHF (Swiss francs; 1 CHF = 1.15 US$ in March 2024).

### Mediators: sleep measures

Baseline sleep quality was quantified based on the validated Pittsburgh Sleep Quality Index (PSQI)—a 19-item questionnaire evaluating sleep patterns over the previous month [30]. The PSQI includes seven components of sleep, including sleep quality, latency, efficiency, duration, disturbances, daytime dysfunction, and use of sleep medications/aids, with each scored from 0 to 3. The overall PSQI score was obtained by summing these scores and the final score ranged from 0 to 21, with higher scores indicating poorer sleep quality. We also created a binary sleep quality variable by using a PSQI score cut-off point of >5 as ‘poor sleep quality’—a value that reflected clinically significant sleep disturbances with sensitivity and specificity of >85% [30–33]. Sleep duration was self-reported as the average number of hours of sleep per night and categorized using physiological cut-offs into short (<6 h/night), recommended or normal (6–8.5 h/night), and long (>8.5 h/night) [34, 35].

### Outcome: pain measures

The presence of pain, chronic pain, and pain severity were assessed at follow-up by using a self-administered questionnaire involving an 11-item pain inventory previously validated in a French population [36]. The presence of pain was measured by using the question: ‘Currently, do you suffer from one or more pains felt every day? (yes/no).’ Participants who reported experiencing pain were asked: ‘How long have you had pain that you feel every day? (<3 months/≥3 months).’ We defined chronic pain as pain lasting for ≥3 months and coded as ‘yes’ versus ‘no’ (no pain or pain lasting for <3 months). Among participants with pain, the pain severity was evaluated by using three questions from the Brief Pain Inventory on a validated numeric rating scale ranging from 0 = no to 10 = worst imaginable pain; participants reported whether pain varied in severity during the day and indicated the highest, lowest, and average pain severity during the last week [37]. The mean pain severity score was the mean of these values.

### Covariates

**Figure 1.**
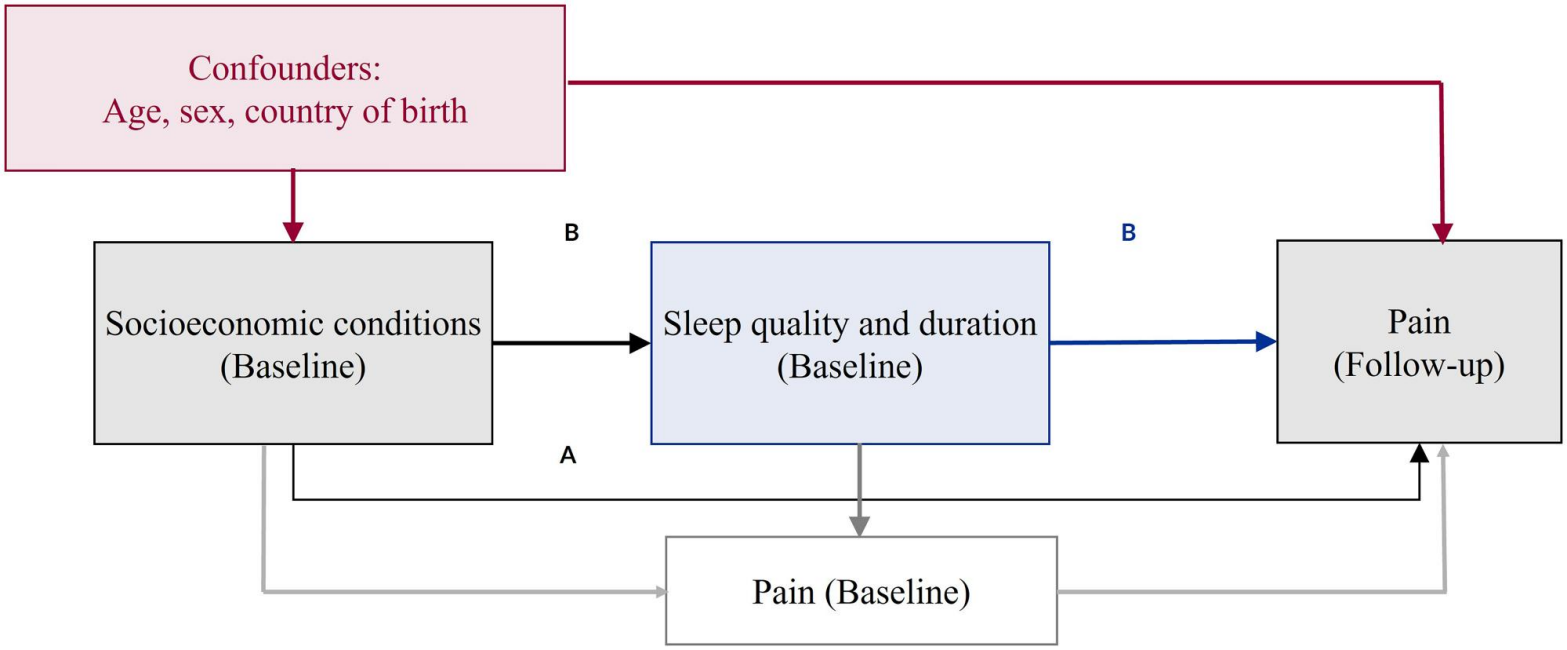
Causal structure for association between socio-economic indicators and pain, mediated by sleep quality and duration. A: Direct causal effect and B: indirect causal effect of socio-economic conditions on pain. Socio-economic conditions include father’s occupation, educational level, occupational position, and income.

Potential confounders were selected based on known associations with the exposure, mediators, and outcomes, and those that did not lie on the causal path, identified by using a directed acyclic graph (Fig. 1). We included age (continuous), sex (men/women), and country of birth (Swiss-born/non-Swiss-born) as potential confounders.

### Statistical analyses

We used linear regression for continuous dependent variables (sleep quality, pain severity score), logistic regression for binary dependent variables (pain, chronic pain, sleep quality), and multinomial logistic regression for the categorical dependent variable of sleep duration. All estimates [odds ratios (ORs) and beta coefficients with 95% confidence intervals (CIs)] were adjusted for age, sex, and country of birth. First, we analysed associations between baseline socio-economic conditions and follow-up pain; second, those between baseline socio-economic conditions and sleep; and third, those between baseline sleep and follow-up pain. Socio-economic indicators were modelled as ordered variables, hypothesizing a linear effect on the outcome [38, 39]. A Cochran–Armitage test showed no departure from the linear trend. A priori, a Wald test provided no evidence of effect modification by age and sex.

We used counterfactual mediation methods to examine the extent to which the association between each socio-economic indicator and pain outcomes was mediated by sleep measures that fulfilled the criteria for mediation (based on the previously mentioned three sets of analyses) (Fig. 1). Counterfactual mediation analysis enables the decomposition of the total effect of socio-economic conditions on pain into direct and indirect components, even in the presence of interactions and non-linearities, overcoming limitations of traditional approaches (e.g. difference-of-coefficients or product-of-coefficients methods) [40, 41]. For counterfactual mediation, two regression models were fitted: (i) a model that predicts pain outcomes based on socio-economic indicators, sleep measures, an interaction term between them, and confounders; and (ii) a model that predicts sleep measures based on socio-economic indicators and confounders. Regression coefficients from these models were used to estimate the NDE (natural direct effect—the effect of socio-economic conditions on pain via pathways that do not involve sleep measures), NIE (natural indirect effect—the effect of socio-economic conditions on pain operating through sleep measures), and MTE (marginal total effect—the total effect of socio-economic conditions on pain) [38, 42]. Subsequently, the proportion of the association between socio-economic conditions and pain that is mediated by sleep was computed (PM: proportion mediated). Confidence intervals were computed through percentiles from bootstrapping with 10 000 simulations [43]. We use the term ‘effects’ as commonly used in mediation research; however, this does not imply that causality can be inferred from our findings.

A priori, we decided not to adjust for baseline pain, as it is a mediator in the causal pathway between socio-economic conditions and follow-up pain (Fig. 1) and such adjustment would have introduced overadjustment or collider bias, leading to biased estimates [44–46]. To show this issue, in analyses of baseline socio-economic conditions or sleep with follow-up pain, we additionally adjusted for baseline pain and compared the results with those of the main analyses. We also did not adjust for other pain risk factors, including psychological factors and health-related behaviours, as these also lie on the causal pathway between socio-economic conditions and pain [8, 44, 47]. Analyses were undertaken by using Stata 16 (StataCorp, College Station, TX, USA).

## Results

Our analytical sample included 1719 participants [52.0% women; mean age (SD) = 60.1 (9.1) years] with a mean (SD) follow-up of 3.8 (0.4) years (Table 1). Most participants reported their father’s occupation as intermediate (23%) or low (54%) and their educational level as intermediate (66%) or high (26%). Approximately a quarter of participants had a low occupational position (26%) and 17% reported income levels of ≤5000 CHF. The mean PSQI score was 4.1 (SD = 2.8) and 44% had a PSQI score of ≥5. Normal sleep duration was reported by 86% of the participants, while 5% had long and 9% had short sleep durations. At follow-up, 47% reported having pain and 40.8% reported having chronic pain. Among those with pain, the mean pain severity score was 3.6 (SD = 1.7).

**Table 1.** Baseline characteristics of included participants.

Characteristics	All (*n* = 1719)
**Demographic characteristics**	
Age (years) (mean ± SD)	60.1 ± 9.1
Men [*n* (%)]	831 (48)
Swiss-born [*n* (%)]	1191 (69)
**Socio-economic indicators [*n* (%)]**	
Father’s occupational position^a^	
High	348 (20)
Intermediate	398 (23)
Low	925 (54)
Educational level	
High	452 (26)
Intermediate	1124 (66)
Low	143 (8)
Occupational position^a^	
High	201 (12)
Intermediate	425 (25)
Low	440 (26)
Household income (CHF)^a^	
>13 000	273 (16)
9500–13 000	313 (18)
7000–9500	327 (19)
5000–7000	350 (20)
3000–5000	225 (13)
Up to 3000	68 (4)
**Sleep measures [*n* (%)]**	
PSQI (continuous; mean ± SD)	4.8 (3.1)
Sleep quality	
Low (PSQI score 0–4)	960 (56)
High (PSQI score ≥5)	759 (44)
Sleep duration (hours)	
Normal (6–8.5)	1480 (86)
Long (>8.5)	92 (5)
Short (<6)	147 (9)

CHF: Swiss Francs; PSQI: Pittsburgh Sleep Quality Index.

a Due to missing data, we excluded 48 participants from analyses including father’s occupation and 163 participants from analyses considering income. For analyses that considered occupational position, only 1065 participants who were working at baseline were included. Thus, percentages do not add up to 100%.

Individuals with low socio-economic conditions were more likely to report pain and have worse pain severity compared with more socio-economically advantaged individuals (Supplementary Table 2). Low occupational position and household income were also associated with chronic pain, whereas father’s occupational position and education showed no such associations. These effects were attenuated after further adjustment for baseline pain, highlighting the potential introduction of overadjustment bias (Supplementary Table 3).

Low socio-economic conditions, across all four indicators, were associated with continuous PSQI scores; however, only household income was associated with low sleep quality when using the PSQI score as a binary variable (Table 2). While no associations were evident between the father’s occupational position and sleep duration, strong associations were observed between low education, household income, and short and long sleep durations. Low occupational position was also associated with short sleep duration.

**Table 2.** Regression models for the associations between socio-economic indicators and sleep indicators.

**PSQI (continuous)** ^a^	*N*	*β* (95% CI)
Father’s occupation	1671	**0.46 (0.14–0.78)**
Education	1719	**0.70 (0.23–1.17)**
Own occupational position	1066	**0.63 (0.21–1.06)**
Household income	1556	**1.34 (0.84–1.84)**
**Sleep quality (PSQI binary)** ^b^	***N***	**OR (95% CI)**
Father’s occupation	1671	1.17 (0.92–1.50)
Education	1719	1.25 (0.88–1.77)
Own occupational position	1066	1.27 (0.90–1.79)
Household income	1556	**2.13 (1.46–3.11)**
**Sleep duration** ^c^	***N***	**OR (95% CI)**
Father’s occupation	1671	
Short sleep (<6 h)		1.16 (0.75–1.81)
Normal sleep		1 (Ref.)
Long sleep (>8.5 h)		1.00 (0.58–1.72)
Education	1719	
Short sleep (<6 h)		**2.11 (1.15–3.88)**
Normal sleep		1 (Ref.)
Long sleep (>8.5 h)		**2.67 (1.21–5.92)**
Own occupational position	1066	
Short sleep (<6 h)		**2.02 (1.08–3.79)**
Normal sleep		1 (Ref.)
Long sleep (>8.5 h)		1.58 (0.54–4.63)
Household income	1556	
Short sleep (<6 h)		**3.67 (1.88–7.15)**
Normal sleep		1 (Ref.)
Long sleep (>8.5 h)		**2.33 (1.00–5.46)**

Examining the association at baseline between socio-economic conditions (exposure variables) and sleep quality and duration (mediators) as one of the mandatory steps while conducting mediation analysis. Bold text indicates statistical significance. PSQI: Pittsburgh Sleep Quality Index.

a Linear regression models for the association between socio-economic indicators (lowest vs. highest) and PSQI score at baseline, adjusting for age, sex, and country of birth.

b Logistic regression models for the association between socio-economic indicators (lowest vs. highest) and sleep quality at baseline, adjusting for age, sex, and country of birth.

c Multinomial logistic regression models for the association between socio-economic indicators (lowest vs. highest) and sleep duration at baseline, adjusting for age, sex, and country of birth.

Both the PSQI score and poor sleep quality were associated with all pain outcomes, while short sleep duration was associated with pain and chronic pain and not pain severity (Table 3). These associations were attenuated after further adjustment for baseline pain (Supplementary Table 4).

**Table 3.** Associations between sleep quality and duration at baseline and pain at follow-up.

Self-reported pain^a^ (*n* = 1719)	OR (95% CI)
PSQI	**1.14 (1.10–1.18)**
Sleep quality (PSQI binary)	**1.91 (1.57–2.33)**
Sleep duration	
Short sleep (<6 h)	**1.86 (1.31–2.64)**
Normal sleep	1 (Ref)
Long sleep (>8.5 h)	1.07 (0.70–1.65)
**Chronic pain^b^ (>3 m vs. no pain or <3 m) (*n* = 1687)**	**OR (95% CI)**
PSQI	**1.13 (1.09–1.18)**
Sleep quality (PSQI binary)	**1.86 (1.52–2.27)**
Sleep duration	
Short sleep (<6 h)	**1.84 (1.29–2.62)**
Normal sleep	1 (Ref.)
Long sleep (>8.5 h)	1.22 (0.79–1.88)
**Pain severity score^c^ (*n* = 699)**	***β* (95% CI)**
PSQI	**0.11 (0.07–0.15)**
Sleep quality (PSQI binary)	**0.48 (0.24–0.73)**
Sleep duration	
Short sleep (<6 h)	0.33 (–0.06 to 0.72)
Normal sleep	1 (Ref)
Long sleep (>8.5 h)	0.46 (–0.04 to 0.96)

Examining the association between sleep quality and duration (mediators) at baseline and pain measures (outcomes) at follow-up as one of the mandatory steps while conducting mediation analysis. Bold text indicates statistical significance. PSQI: Pittsburgh Sleep Quality Index.

a Logistic regression models for the association between sleep indicators at baseline and everyday pain at follow-up, adjusting for age, sex, and country of birth.

b Logistic regression models for the association between sleep indicators at baseline and chronic pain at follow-up, adjusting for age, sex, and country of birth.

c Linear regression models for the association between sleep indicators at baseline and pain severity score at follow-up, adjusting for age, sex, and country of birth.

Based on the results from the previous sets of analyses, counterfactual mediation was conducted where socio-economic indicators were associated with pain outcomes, with sleep measures, and where sleep measures were associated with pain outcomes. Thus, Table 4 presents counterfactual mediation models for associations between socio-economic indicators and pain, chronic pain, and pain severity, mediated by the PSQI score (continuous) or short sleep duration. Low educational level (OR_MTE_ 1.49; 95% CI: 1.00–2.18), occupational position (1.63; 95% CI: 1.19–2.46), and household income (1.71; 95% CI: 1.16–2.56) were associated with pain, with the PSQI score mediating 30%, 21%, and 45% of these associations, respectively. Low occupational position (1.63; 95% CI: 1.11–2.30) and household income (1.63; 95% CI: 1.11–2.69) were associated with chronic pain, with the PSQI score meditating 20% and 51% of these associations, respectively. All socio-economic indicators were associated with pain severity, with the PSQI score mediating 15% of the association with the father’s occupation, 9% with education, and 16% with household income. The MTE of low education on pain was 1.65 (95% CI: 1.05–2.46), with short sleep duration appearing to mediate 31% of the association. The effects of own occupation position and household income on pain were not mediated by short sleep duration. Finally, the MTE for chronic pain was 1.59 (95% CI: 1.07–2.60) for low education and 1.75 (95% CI: 1.15–2.78) for low household income, with short sleep duration mediating these associations by 36% and 37%, respectively. The effect of occupational position and chronic pain was not mediated by short sleep duration. The results of the counterfactual mediation analysis for sleep measures not meeting the mediation criteria are presented in Supplementary Table 3.

**Table 4.** Counterfactual mediation estimates for socio-economic indicators and pain association, mediated by sleep quality and duration.

	Mediating role of PSQI (continuous)				
		MTE	NDE	NIE	PM
	*N*	OR (95% CI)	OR (95% CI)	OR (95% CI)	% (95% CI)
**Self-reported pain^a^**					
Father’s occupation	1671	1.07 (0.82–1.42)	1.00 (0.79–1.33)	**1.06 (1.02–1.13)**	**95 (28–176)**
Education	1719	**1.49 (1.00–2.18)**	1.34 (0.92–1.93)	**1.11 (1.04–1.26)**	**30 (1–135)**
Own occupational position	1066	**1.63 (1.19–2.46)**	**1.50 (1.08–2.15)**	**1.09 (1.03–1.20)**	**21 (7–54)**
Household income	1556	**1.71 (1.16–2.56)**	1.39 (0.93–2.03)	**1.23 (1.10–1.47)**	**45 (25–147)**
**Chronic pain^b^ (>3 m vs. no pain or <3 m)**					
Father’s occupation	1641	1.11 (0.84–1.45)	1.04 (0.80–1.36)	**1.06 (1.01–1.18)**	58 (–26 to 106)
Education	1687	1.39 (0.93–2.12)	1.25 (0.86–1.98)	**1.11 (1.03–1.26)**	35 (–60 to 295)
Own occupational position	1046	**1.63 (1.11–2.30)**	**1.50 (1.04–2.11)**	**1.08 (1.02–1.18)**	**20 (5–61)**
Household income	1533	**1.63 (1.11–2.69)**	1.30 (0.92–2.15)	**1.25 (1.11–1.45)**	**51 (22–152)**
	
	***N***	***β* (95% CI)**	***β* (95% CI)**	***β* (95% CI)**	**% (95% CI)**
	
**Pain severity score^c^**					
Father’s occupation	677	**0.39 (0.04–0.72)**	**0.33 (0.00–0.66)**	**0.06 (0.00–0.14)**	**15 (1–93)**
Education	699	**1.31 (0.87–1.69)**	**1.19 (0.69–1.54)**	**0.12 (0.01–0.28)**	**9 (1–24)**
Own occupational position	398	**0.83 (0.41–1.28)**	**0.79 (0.37–1.22)**	0.04 (–0.04 to 0.17)	5 (–6 to 21)
Household income	627	**1.22 (0.75–1.65)**	**1.02 (0.53–1.48)**	**0.20 (0.05–0.43)**	**16 (3–37)**

	**Mediating role of short sleep duration**				
	
		**MTE**	**NDE**	**NIE**	**PM**
	***N***	**OR (95% CI)**	**OR (95% CI)**	**OR (95% CI)**	**% (95% CI)**

**Self-reported pain^d^**					
Father’s occupation	1671	1.08 (0.85–1.40)	1.07 (0.85–1.44)	1.01 (0.98–1.05)	11 (–9 to 141)
Education	1719	**1.65 (1.05–2.46)**	**1.45 (0.94–2.02)**	**1.14 (1.02–1.53)**	**31 (5–94)**
Own occupational position	1066	**1.53 (1.10–2.28)**	**1.51 (1.09–2.23)**	1.02 (0.99–1.08)	4 (–4 to 34)
Household income	1556	**1.68 (1.11–2.38)**	**1.55 (1.04–2.18)**	1.08 (0.98–1.33)	19 (–6 to 64)
**Chronic pain^e^ (>3 m vs. no pain or <3 m)**					
Father’s occupation	1641	1.15 (0.86–1.50)	1.14 (0.86–1.46)	1.00 (0.99–1.05)	6 (–9 to 227)
Education	1687	**1.59 (1.07–2.60)**	1.37 (0.95–2.12)	**1.16 (1.02–1.43)**	**36 (1–120)**
Own occupational position	1046	**1.51 (1.02–2.10)**	**1.48 (1.00–2.02)**	1.01 (0.99–1.09)	5 (–3 to 66)
Household income	1533	**1.75 (1.15–2.78)**	**1.47 (1.01–2.38)**	**1.19 (1.02–1.52)**	**37 (5–88)**

PSQI: Pittsburgh Sleep Quality Index; MTE: marginal total effect; NDE: natural direct effect; NIE: natural indirect effect; PM: proportion mediated. Bold text indicates statistical significance.

a Association between socio-economic indicators (lowest vs. highest) and PSQI score at baseline and everyday pain at follow-up, adjusting for sex, age, and country of birth.

b Association between socio-economic indicators (lowest vs. highest) and PSQI score at baseline and chronic pain at follow-up, adjusting for sex, age, and country of birth.

c Association between socio-economic indicators (lowest vs. highest) and PSQI score at baseline and pain severity score at follow-up, adjusting for sex, age, and country of birth.

d Association between socio-economic indicators (lowest vs. highest) and short sleep duration at baseline and everyday pain at follow-up, adjusting for sex, age, and country of birth.

e Association between socio-economic indicators (lowest vs. highest) and short sleep duration at baseline and chronic pain at follow-up, adjusting for sex, age, and country of birth.

## Discussion

Using counterfactual-based mediation analysis, we showed that sleep quality as measured by using the PSQI score mediated socio-economic inequities in pain, chronic pain, and pain severity scores. Although uncertainty around the percentage mediated was high, sleep quality explained 30% of the educational, 21% of the occupational, and 45% of the income inequities in pain; 20% of the occupation and 51% of the income inequities in chronic pain; and 15%, 9%, and 16% of the father’s occupation, educational, and income inequities in pain severity, respectively. Additionally, we found that short sleep duration explained 31% of the association between education and pain as well as 36%–37% of both the education and income inequities in chronic pain.

A large body of evidence suggests an association between different socio-economic indicators and pain experience and severity, aligning with our findings [8, 48]. All socio-economic indicators were associated with a continuous PSQI score, but not with a binary variable for overall sleep quality when using a PSQI-score cut-off of 5 (except for income). Despite this cut-off showing high sensitivity (89.6%) and specificity (86.5%) for identifying sleep disorders, a systematic review found that its validity varies substantially across clinical and non-clinical samples [49]. We also found strong associations between education, occupational position, and household income with suboptimal sleep duration, with stronger associations for short sleep than for long sleep, consistently with previous studies [20–24, 34, 35, 50]. Low socio-economic conditions are linked to financial hardship, job-related psychosocial stress, shift work, poor living conditions, lack of social support, and loneliness, leading to chronic and mental stress, which in turn negatively influences both sleep quality and quantity [34, 50].

Our results also confirm the association between sleep quality and pain outcomes, aligning with results from a systematic review of longitudinal and experimental studies [18]. We also found that short, but not long, sleep duration is associated with increased odds of pain experience and chronic pain but not with pain severity. Previous studies have reported associations between short and long sleep durations with different pain measures, with greater support for short sleep duration impacting pain than long sleep duration [18, 51]. Short sleep duration may be a result of complex social, behavioural, and/or physiological conditions. In lower socio-economic groups, longer working hours, greater job demands, and shift work may contribute to shorter sleep duration [35]. Physiological mechanisms linking sleep quality and quantity to pain include elevated pro-inflammatory markers, alterations in the function of dopamine, and changes in psychological affects (e.g. moods, anxiety) [18, 52]

Considering the mediating role of sleep measures, the only two available studies examined neighbourhood-level socio-economic inequities in pain and reported mixed results [12, 27]. No prior studies have examined the mediating role of sleep quality and duration on the association between individual-level socio-economic indicators and pain measures. We found that the association between adulthood socio-economic conditions and pain experience was substantially mediated (19%–51%) by sleep quality (continuous PSQI scores). Similar results were found for chronic pain, with PSQI scores mediating the occupation and income inequities in pain by 20% and 51%, respectively. The mediation role of sleep quality and short sleep duration was greatest for the association between income and pain, likely because the association with income was the strongest compared with other socio-economic indicators. Financial hardship may contribute to stress, anxiety, uncertainty, and neighbourhood conditions (e.g. noise), impacting sleep [53, 54]. The results support the need to address sleep inequities, especially income-related. Of note, short sleep duration mediated the education inequities in pain by 31% and education and income inequities in chronic pain by 37%, but showed no mediation when other socio-economic indicators were used. These results highlight that sleep quality and short sleep duration partially mediate the socio-economic inequities in specific pain outcomes.

## Strength and limitations

A main strength of our study includes using four different indicators to capture socio-economic conditions across the life course, measuring both sleep quality and quantity, and measuring pain in terms of chronicity and severity. The counterfactual mediation method enabled us to more accurately examine the mediating role of sleep compared with traditional methods [55].

There are also important limitations. The CoLaus|PsyCoLaus comprises individuals of European origin living in a wealthy country, likely contributing to relatively low exposure to adverse socio-economic conditions. While the PSQI has been validated across populations, it has not been validated in a Swiss population. Our analysis included participants with and without baseline pain; given that pain is a common issue among adults and older adults, excluding participants with pain limits generalizability and substantially reduces statistical power [56]. The relatively small sample size for pain severity analyses also limited the statistical power to detect smaller effects. Our findings may be subject to selection bias and limited generalizability, as analyses were restricted to participants with complete baseline sleep and follow-up pain data, possibly underestimating inequities. Missing data on own occupational position were extensive and those with incomplete data tended to have lower educational and income levels, suggesting that associations with pain outcomes and the mediating role of sleep measures may have been underestimated. The lack of mother’s occupational data is another limitation, although, given the cohort’s age and historical context, the father’s occupation likely captured much of the early-life socio-economic conditions. The father’s occupation and education were retrospectively reported, capturing early-life socio-economic conditions with a strong theoretical temporal link with adulthood sleep measures. However, individual occupation position and income were measured concurrently with sleep, limiting temporal inference [57]. The lack of longitudinal early-life data also prevents ruling out reverse causation.

Our analysis relies upon strong assumptions of no unmeasured confounding between the exposure, mediators, and outcomes. While we adjusted for several confounders based on prior research on health inequities, unmeasured confounding remains possible. Potential unmeasured confounders include neighbourhood characteristics, job conditions, and early-life health, and mediator–outcome confounders (i.e. behavioural and psychosocial factors) [8] not assessed in this study. These sources of residual confounding could bias estimates in either direction, with underestimation or overestimation of the observed associations. Proper adjustment for mediator–outcome confounders affected by the exposure would require advanced modelling beyond the scope of this study. Further research is therefore needed to better understand the behavioural and physiological mechanisms underlying pain exacerbation in a detrimental socio-economic context.

## Conclusions

In this study, we found that sleep quality and short sleep duration partially mediate the socio-economic inequities in pain, chronic pain, and pain severity. The results from this study support early intervention in sleep quality and duration, where indicated, among socio-economically disadvantaged individuals as part of an integrated approach to reducing pain inequities. Beyond targeted sleep interventions, addressing broader structural drivers—such as education, income security, and working conditions—may have a wider and more sustained impact on reducing inequities in pain.

## Ethics approval

The institutional Ethics Committee of the University of Lausanne approved the CoLaus|PsyCoLaus study (reference 16/03). The study was performed in agreement with the Helsinki Declaration and its former amendments, and in accordance with the applicable Swiss legislation. All participants gave their signed informed consent before entering the study.

## Supplementary Material

dyaf176_Supplementary_Data

## Data Availability

The data of the CoLaus|PsyCoLaus study used in this article cannot be fully shared, as they contain potentially sensitive personal information on participants. According to the Ethics Committee for Research of the Canton of Vaud, sharing these data would be a violation of the Swiss legislation with respect to privacy protection. However, coded individual-level data that do not allow researchers to identify participants are available on request for researchers who meet the criteria for data sharing of the CoLaus|PsyCoLaus Datacenter (CHUV, Lausanne, Switzerland). Any researcher affiliated to a public or private research institution who complies with the CoLaus|PsyCoLaus standards can submit a research application to research.colaus@chuv.ch or research.psycolaus@chuv.ch. Proposals requiring baseline data only will be evaluated by the baseline (local) Scientific Committee (SC) of the CoLaus and PsyCoLaus studies. Proposals requiring follow-up data will be evaluated by the follow-up (multicentric) SC of the CoLaus|PsyCoLaus cohort study. Detailed instructions for gaining access to the CoLaus|PsyCoLaus data used in this study are available at www.colaus-psycolaus.ch/professionals/how-to-collaborate/.
