# Supplementary material for: Mediating impact of sleep quality and duration on the relationship between socio-economic conditions and pain
Source: Int J Epidemiol. 2025 Oct 30;54(6):dyaf176. doi: 10.1093/ije/dyaf176 (PMC12576963; doi:10.1093/ije/dyaf176)
Supplement: dyaf176_Supplementary_Data [file dyaf176_supplementary_data.docx]

**Supplementary File**

**Table S1**. Sample size and exclusion criteria for different analyses

| **Pain measure** | **Socio-economic indicator** | **Sample size** | **Exclusion/inclusion criteria** |
| --- | --- | --- | --- |
| Pain |  | 1719 | Those with baseline sleep measures and pain measures at follow-up |
|  | Father’s occupation | 1671 | N=48 participants missing father’s occupation data were excluded |
|  | Education | 1719 | - |
|  | Occupation | 1066 | Those who were working at baseline were included |
|  | Income | 1566 | N=163 participants with missing income data were excluded |
| Chronic pain |  | 1687 | N=32 participants with missing chronic pain data were excluded |
|  | Father’s occupation | 1641 | N=46 participants missing father’s occupation data were excluded |
|  | Education | 1687 | - |
|  | Occupation | 1046 | Those who were working at baseline were included |
|  | Income | 1533 | N=154 participants with missing income data were excluded |
| Pain severity |  | 699 | Participants who reported experiencing pain and had pain severity data at follow-up |
|  | Father’s occupation | 677 | N=22 participants missing father’s occupation data were excluded |
|  | Education | 699 | - |
|  | Occupation | 398 | Those who were working at baseline were included |
|  | Income | 627 | N=72 participants with missing income data were excluded |

**Table S2**. Regression models for the associations between indicators of socio-economic conditions across the life course at baseline and pain scores at follow-up

| **Self-reported pain**^a^ | **N** | **OR [95%CI]** |
| --- | --- | --- |
| Father’s occupation | 1671 | 1.05 [0.82;1.33] |
| Education | 1719 | **1.42 [1.00;2.01]** |
| Own occupational position | 1066 | **1.58 [1.12;2.23]** |
| Household income | 1556 | **1.57 [1.08;2.28]** |
| **Chronic pain**^b^ **(>3m vs. no pain or <3m)** |  |  |
| Father’s occupation | 1641 | 1.09 [0.85;1.41] |
| Education | 1687 | 1.31 [0.91;1.87] |
| Own occupational position | 1046 | **1.54 [1.07;2.21]** |
| Household income | 1533 | **1.49 [1.01;2.18]** |
| **Pain severity score**^c^ | **N** | **β [95%CI]** |
| Father’s occupation | 677 | **0.39 [0.08;0.70]** |
| Education | 699 | **1.31 [0.85;1.76]** |
| Own occupational position | 398 | **0.83 [0.39;1.25]** |
| Household income | 627 | **1.22 [0.76;1.69]** |

Examining the association between socio-economic conditions (exposure variables) at baseline and pain measures (outcomes) at follow-up as one of the mandatory steps while conducting mediation analysis.

Abbreviations: CI, confidence interval.

Bold text indicates statistical significance.

^a^ Logistic regression models for the association between socio-economic indicators at baseline (lowest vs. highest) and everyday pain at follow-up, adjusting for age, sex, country of birth.

^b^ Logistic regression models for the association between socio-economic indicators at baseline (lowest vs. highest) and chronic pain at follow-up (>3 months vs. no pain or <3 months), adjusting for age, sex, country of birth.

^c^ Linear regression models for the association between socio-economic indicators at baseline (lowest vs. highest) and Pain severity score at follow-up, adjusting for age, sex, country of birth.

**Table S3**. Regression models for the associations between indicators of socio-economic conditions across the life course at baseline and pain scores at follow-up – explanatory analysis further adjusting for baseline pain

| **Self-reported pain**^a^ | **N** | **OR [95%CI]** |
| --- | --- | --- |
| Father’s occupation | 1631 | 1.10 [0.84;1.45] |
| Education | 1679 | 1.34 [0.91;1.99] |
| Own occupational position | 1042 | 1.45 [0.99;2.11] |
| Household income | 1519 | 1.36 [0.89;2.07] |
| **Chronic pain**^b^ **(>3m vs. no pain or <3m)** |  |  |
| Father’s occupation | 1602 | 1.13 [0.85;1.49] |
| Education | 1648 | 1.26 [0.84;1.89] |
| Own occupational position | 1023 | 1.42 [0.96;2.10] |
| Household income | 1497 | 1.26 [0.82;1.93] |
| **Pain severity score**^c^ | **N** | **β [95%CI]** |
| Father’s occupation | 664 | **0.40 [0.09;0.71]** |
| Education | 686 | **1.27 [0.82;1.72]** |
| Own occupational position | 390 | **0.74 [0.31;1.16]** |
| Household income | 615 | **1.16 [0.70;1.63]** |

Examining the association between socio-economic conditions (exposure variables) at baseline and pain measures (outcomes) at follow-up as one of the mandatory steps while conducting mediation analysis.

Abbreviations: CI, confidence interval.

Bold text indicates statistical significance.

^a^ Logistic regression models for the association between socio-economic indicators at baseline (lowest vs. highest) and everyday pain at follow-up, adjusting for age, sex, country of birth as well as baseline pain.

^b^ Logistic regression models for the association between socio-economic indicators at baseline (lowest vs. highest) and chronic pain at follow-up (>3 months vs. no pain or <3 months), adjusting for age, sex, country of birth as well as baseline pain.

^c^ Linear regression models for the association between socio-economic indicators at baseline (lowest vs. highest) and Pain severity score at follow-up, adjusting for age, sex, country of birth as well as baseline pain.

**Table S4.** Associations between sleep quality and duration at baseline and pain at follow-up– explanatory analysis further adjusting for baseline pain

| **Self-reported pain**^a^ **(n=1679)** | ***OR [95%CI]*** |
| --- | --- |
| PSQI | **1.09 [1.05;1.13]** |
| Sleep quality (PSQI binary) | **1.58 [1.17;2.12]** |
| Sleep duration |  |
| Short sleep (<6h) | **1.56 [1.05;2.31]** |
| Normal sleep | 1 (Ref) |
| Long sleep (>8.5h) | 1.11 [0.69;1.78] |
| **Chronic pain**^b^ **(>3m vs. no pain or <3m) (n=1648)** | ***OR [95%CI]*** |
| PSQI | **1.08 [1.04;1.12]** |
| Sleep quality (PSQI binary) | **1.51 [1.12;2.03]** |
| Sleep duration |  |
| Short sleep (<6h) | **1.58 [1.07;2.34]** |
| Normal sleep | 1 (Ref) |
| Long sleep (>8.5h) | 1.30 [0.81; 2.08] |
| **Pain severity score**^c^ **(n=686)** | ***β [95%CI]*** |
| PSQI | **0.10 [0.06;0.13]** |
| Sleep quality (PSQI binary) | 0.27 [-0.03;0.57] |
| Sleep duration |  |
| Short sleep (<6h) | 0.32 [-0.07;0.70] |
| Normal sleep | 1 (Ref) |
| Long sleep (>8.5h) | **0.51 [0.01;1.01]** |

Examining the association between sleep quality and duration (mediators) at baseline and pain measures (outcomes) at follow-up as one of the mandatory steps while conducting mediation analysis.

PSQI, Pittsburgh Sleep Quality Index.

Bold text indicates statistical significance.

^a^ Logistic regression models for the association between sleep indicators at baseline and everyday pain at follow-up, adjusting for age, sex, country of birth as well as baseline pain.

^b^ Logistic regression models for the association between sleep indicators at baseline and chronic pain at follow-up, adjusting for age, sex, country of birth as well as baseline pain.

^c^ Linear regression models for the association between sleep indicators at baseline and Pain severity score at follow-up, adjusting for age, sex, country of birth as well as baseline pain.

**Table S5.** Counterfactual mediation estimates for the association between socio-economic indicators and pain measures, mediated by PSQI.

|  | **Mediating role of PSQI (binary)** | | | |  |
| --- | --- | --- | --- | --- | --- |
|  |  | **MTE** | **NDE** | **NIE** | **PM** |
|  | **N** | **OR [95% CI]** | **OR [95% CI]** | **OR [95%CI]** | **% [95% CI]** |
| **Self-reported pain**^a^ |  |  |  |  |  |
| Father's occupation | 1671 | 1.07 [0.84;1.40] | 1.04 [0.82;1.35] | 1.02 [0.98;1.07] | ~~-~~ |
| Education | 1719 | **1.53 [1.00;2.19]** | 1.45 [0.97;2.05] | 1.04 [0.97;1.13] | ~~-~~ |
| Own occupational position | 1066 | **1.59 [1.17;2.38]** | **1.53 [1.11;2.16]** | 1.03 [0.99;1.12] | ~~-~~ |
| Household income | 1556 | **1.74 [1.17;2.56]** | 1.47 [0.99;2.08] | **1.18 [1.08;1.34]** | **36 [18;101]** |
|  |  |  |  |  |  |
| **Chronic pain**^b^ **(>3m vs. no pain or <3m)** | |  |  |  |  |
| Father's occupation | 1641 | 1.12 [0.87;1.49] | 1.09 [0.84; 1.42] | 1.02 [0.99;1.08] | ~~-~~ |
| Education | 1687 | 1.43 [0.98;2.22] | 1.36 [0.95;2.11] | 1.04 [0.97;1.14] | ~~-~~ |
| Own occupational position | 1046 | **1.60 [1.10;2.24]** | **1.54 [1.06;2.11]** | 1.03 [0.98;1.12] | ~~-~~ |
| Household income | 1533 | **1.71 [1.15;2.80]** | 1.39 [0.96;2.21] | **1.23 [1.10;1.41]** | **44 [16;113]** |

Abbreviations: MTE, marginal total effect; NDE, natural direct effect; NIE, natural indirect effect; PM, proportion mediated.

Bold text indicates statistical significance.

^a^ Association between socio-economic indicators (lowest vs. highest) and PSQI at baseline and everyday pain at follow-up adjusting for sex, age, country of birth.

^b^ Association between socio-economic indicators (lowest vs. highest) and PSQI at baseline and chronic pain at follow-up adjusting for sex, age, country of birth.

**Figure S1** Flow diagram of CoLaus|PsyColaus study included participants.

2009

2012

2017

2014

Participants with sleep data

N = 2586

Participants with pain data

N = 1719

2^nd^ follow-up

N = 4881

1^st^ follow-up

N = 5064
